# Skin infections in Australian Aboriginal children: a narrative review

**DOI:** 10.5694/mja2.50361

**Published:** 2019-10-20

**Authors:** Lucy Davidson, Jessica Knight, Asha C Bowen

**Affiliations:** ^1^ University of Western Australia Perth WA; ^2^ Wesfarmers Centre for Vaccines and Infectious Diseases Telethon Kids Institute Perth WA; ^3^ Perth Children's Hospital Perth WA

**Keywords:** Skin diseases, infectious, Streptococcus, Staphylococcus, Cellulitis, Parasitic diseases, Social determinants of health

## Abstract

- Impetigo, scabies, cellulitis and abscesses are common in Australian Aboriginal children. These conditions adversely affect wellbeing and are associated with serious long term sequelae, including invasive infection and post‐infectious complications, such as acute post‐streptococcal glomerulonephritis and acute rheumatic fever, which occurs at the highest documented rates in the world in remote Aboriginal communities.
- Observational research in remote communities in northern Australia has demonstrated a high concurrent burden of scabies and impetigo and their post‐infectious complications.
- Few data are available for other Australian states, especially for urban Aboriginal children; however, nationwide hospital data indicate that the disparity between Aboriginal and non‐Aboriginal children in skin infection prevalence also exists in urban settings.
- The Australian *National Healthy Skin Guideline* summarises evidence‐based treatment of impetigo, scabies and fungal infections in high burden settings such as remote Aboriginal communities. It recommends systemic antibiotics for children with impetigo, and either topical permethrin or oral ivermectin (second line) for the individual and their contacts as equally efficacious treatments for scabies. β‐Lactams are the treatment of choice and trimethoprim–sulfamethoxazole and clindamycin are effective alternatives for treatment of paediatric cellulitis. Abscesses require incision and drainage and a 5‐day course of trimethoprim–sulfamethoxazole or clindamycin.
- Addressing normalisation of skin infections and the social determinants of skin health are key challenges for the clinician. Research is underway on community‐wide skin health programs and the role for mass drug administration which will guide future management of these common, treatable diseases.

The skin is the largest organ of the body and is commonly visible, particularly on the arms and legs of children. As such, skin infections have an impact on overall health and on self‐image and wellbeing. Impetigo, scabies, cellulitis and abscesses are very common in Australian Aboriginal or Torres Strait Islander (respectfully referred to hereafter as Aboriginal) children (Box 1).^4^, ^5^ This review provides an update on the burden, serious sequelae and treatment of these skin infections.

Box 1 **Questions for future research**

Future research to improve skin health for Australian Aboriginal children must include sustainable, community‐wide strategies for impetigo and scabies that address the diagnosis, treatment and prevention of skin infections in an integrated method (SToP trial ANZCTR 12618000520235, starting in the remote Kimberley region, WA, in 2019). Commencing in 2019, research in Fiji will address whether scabies control using mass drug administration of ivermectin will reduce the burden of bacterial skin and soft tissue infection admissions to hospital (ANZCTR 12618000461291). These will be important clinical data to inform the next steps in the Australian context. Recent mass drug administration trials in the Pacific have shown that azithromycin in addition to ivermectin for control of scabies does not have an adjunctive benefit on the burden of impetigo.^1^, ^2^ It remains to be seen whether this is also the case for the remote communities in Australia, where the burden of scabies is lower than that reported in the Pacific.^3^ Further research in partnership with Aboriginal people is needed to understand how traditional Aboriginal knowledge to treat and prevent skin infections can be integrated into these recommendations.

## Definitions

Impetigo, also called skin sores, school sores or pyoderma, is an infection of the superficial skin by *Staphylococcus aureus* and/or *Streptococcus pyogenes*.^6^ Infection may be direct or secondary to cutaneous barrier damage resulting from insect bites, minor trauma, or other pruritic dermatoses, especially scabies, head lice and tinea.^7^ Impetigo begins as erythematous papules, progressing to thinly walled pustules that rupture and crust over with a progressively thickening scab.^8^ As healing begins, the scab shrinks, tethering surrounding skin to heal the eroded base, often without scarring.^6^ Without treatment, resolution can take 30 days,^6^ and infection of all other household members may occur within 21 days^9^ (Box 2).^10^

Box 2 Figure showing a secondarily infected scabies infection (A), a scabies infestation (B),* and purulence, crusting and peeling of impetigo sores (C)

**Figure:**
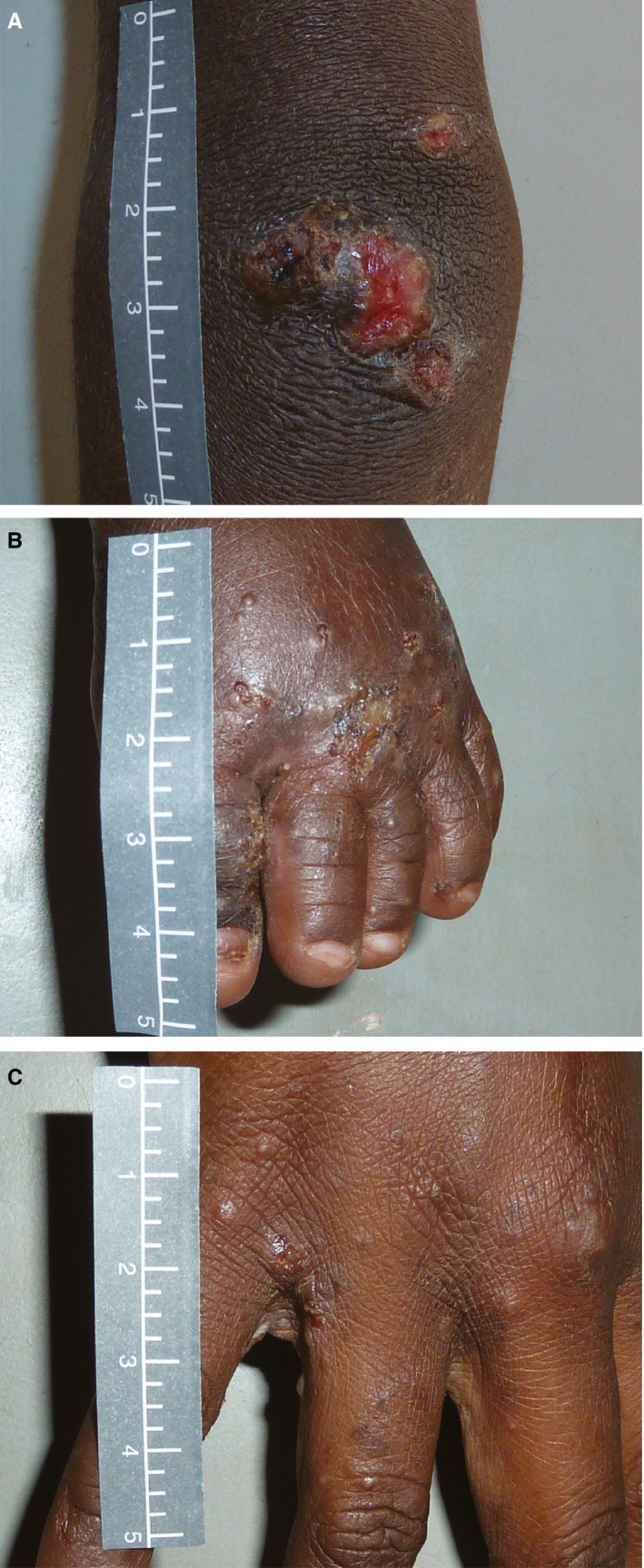
*The papules of 1–2 mm between the fingers are evidence of burrowing scabies mites that are very itchy

Scabies is skin infestation by the mite *Sarcoptes scabeii* var. *hominis*, which provokes an inflammatory response resulting in pruritic skin lesions.^11^ It is a disease of overcrowded housing, not poor hygiene.^12^ Scabies is an important cause of secondary bacterial infection, both due to skin breaks caused by scratching and via mite‐induced reduction in immunity^11^, ^12^ (Box 2).

Erysipelas refers to bacterial infection of the dermis and lymphatics; it is caused by *S. pyogenes* 8 and presents with more clearly delineated borders of inflammation than cellulitis.^8^ Cellulitis involves the deep dermis and subcutaneous tissue, and presents with fever and painful, rapidly spreading erythema of the limbs.^8^ The most common causal pathogen of cellulitis is *S. pyogenes*, but it may also be caused by *S. aureus* or a co‐infection.^13^

An abscess (also known as a boil) is a walled‐off collection of pus in the dermis and deeper skin tissues; it begins as a small lesion and rapidly increases in size, causing fever, pain and erythema. Folliculitis is an infection of a hair follicle causing suppuration of the subcutaneous tissue; multiple contiguous infected hair follicles can form a larger and deeper carbuncle.^8^ Children often present with small to medium‐sized abscesses in the buttocks and lower limbs, usually caused by *S. aureus*.^14^

## Epidemiology of skin infections in Australian Aboriginal children

### Sources and selection criteria for the narrative review

We searched PubMed, MEDLINE, the Cochrane Database of Systematic Reviews and the Grey Literature Report using the search terms “skin sores”, “impetigo”, “pyoderma”, “skin infection”, “cellulitis”, “pyomyositis”, “abscess”, and “burden”, “epidemiology”, and “Aboriginal” or “Torres Strait Islander” or “Indigenous” and “Australia”. We included studies reporting data on Australian Aboriginal children, focusing on systematic reviews and high quality randomised controlled trials published within the past 5 years.

### Global context

Ten observational studies over two decades in remote northern Australia have found that Australian Aboriginal children have the highest documented prevalence of impetigo worldwide.^4^ The median prevalence of impetigo in remote Australian Aboriginal children is 45% (interquartile range [IQR], 34–49%), equating to almost half of all Aboriginal children in remote Australia with impetigo at any one time,^1^ while up to one‐third of children will also have scabies^4^, ^5^ (Box 3). Comparatively, the median prevalence of impetigo in Africa (median, 7.0%; IQR, 4.1–12.3%), Asia (median, 7.3%; IQR 3.0–16.1%) and Oceania (median, 29.7%; IQR, 14.7–42.0%) is much lower, but contributes more substantially to the overall burden of children with impetigo at any one time due to larger populations, being in excess of 162 million children.^4^ Likewise, the prevalence of scabies in remote Aboriginal children (ranging from 16.1% to 35%)^19^, ^21^, ^23^ is the third highest rate documented in any country,^5^ which is noteworthy given that scabies is a common disease worldwide. The Global Burden of Disease estimate for the prevalence of scabies was 204 151.7 cases globally in 2015 (95% CI, 177 533.7–237 466.2).^27^

Box 3 Burden of impetigo and scabies from community‐based prevalence studies of Australian Aboriginal children living in remote areas*

**Table:** 

Study	Year	Region	Number of patients surveyed (age 0–15 years)	Impetigo prevalence (age 0–15 years)†	Scabies prevalence (age 0–15 years)‡
Nimmo et al^15^	1990	QLD	120	43%	nd
Van Buynder et al^16^	1992	NT	180	17%	nd
Streeton et al^17^	1993	QLD	583	34%	nd
Carapetis et al^18^	1994	NT	81	48%	nd
Carapetis et al^19^	1994	NT	62	69%	29%
Shelby‐James et al^20^	1995	NT	79	90%	23%
Wong et al^21^	2000	NT	217	23%	35%
Lehmann et al^22^	2000	WA	121	66%	5%
Andrews et al^23^	2004	NT	582	46%	16%
Andrews et al^23^	2004	NT	2001	40%	nd
Heath et al^24^	2005*	QLD	157	13%	nd
Tasani et al^25^	2012*	NT	1751	50%	14%
Leach et al^26^	2014*	NT	651	18%	12%

nd = no data; NT = Northern Territory; QLD = Queensland; WA = Western Australia.

* Adapted from Bowen et al^4^ and updated with more recent publications.

† Mean (standard deviation [SD]), 42.8 ± 21.8%; median, 43% (interquartile range [IQR], 20.5–50.8%).

‡ Mean (SD), 19.1 ± 9.6%; median 16% (IQR, 12–29%).

### The Australian context

Observational surveillance in the remote Northern Territory since the early 1990s confirm a high burden of skin infections.^7^ In East Arnhem, the Healthy Skin Project demonstrated the ubiquity of skin infections.^23^ Almost every child studied contracted impetigo and scabies at least once during the study.^28^ This dataset also documented the high recurrence rates of the two conditions.^29^ In the first year of life, children presented a median of three times for scabies (IQR, 1–4) and two for impetigo (IQR, 1–5), a significant burden for individuals and communities.^29^ The highest burden is in infancy and the first years of life.^29^

In Western Australia, a study evaluating the benefit of swimming pools in two remote semi‐arid Aboriginal communities documented a skin infection prevalence comparable to the NT, with 62–70% of children studied having impetigo, 8% having abscesses, 25% with fungal infections, and 5% having scabies before the pool opened.^22^ A more recent clinical audit from the Pilbara (WA) of children aged under 5 years showed that these skin infections accounted for the largest proportion of clinic visits (16%) of any reason for attendance.^30^ A novel complementary source of skin epidemiology data comes from children admitted to hospital in WA. More than 50% of all children admitted to hospital in two regional centres studied had a skin infection (49% had impetigo and 8% had scabies), and in these high prevalence settings, health care workers were under‐reporting skin infections.^31^ Linked hospitalisation data for all children born in WA between 1996 and 2012 (*n* = 469 589), of whom 6.7% were Aboriginal, found that hospitalisation rates for skin infections were 15 times higher for Aboriginal children than non‐Aboriginal children (95% CI, 14.5–15.5; *P *< 0.001), and most commonly they were for abscesses (42.2%), cellulitis (26.0%), impetigo (14.3%) and scabies (15.8%).^32^

Three studies reported impetigo prevalence in Queensland,^15^, ^17^, ^24^ ranging from 13% to 43%. Scabies prevalence was not reported. Aboriginal children in Townsville were three times more likely to have skin infections than non‐Aboriginal children.^24^ Ten per cent of all paediatric admissions to Mount Isa Base Hospital were attributable to impetigo and scabies.^33^

Nationwide hospital statistics indicate that skin infections are a problem for urban Aboriginal Australians as well as for those living in remote settings. In the urban setting, Aboriginal children aged under 4 years were twice as likely to be admitted to hospital under the principal diagnosis of “diseases of the skin and subcutaneous tissue” than non‐Aboriginal children.^34^ More research to document the burden of skin infections in Aboriginal children living in urban settings is needed.

Cellulitis has the highest total health and economic burden of all group A *Streptococcus* (GAS) diseases in Australia, accounting for almost half the 23 528 disability‐adjusted life years and the $185.1 million in health care costs attributable to GAS diseases.^35^ Abscess diagnosis is growing, with a 48% increase in yearly hospital admissions in Australia for cutaneous abscesses between 1999–2000 and 2007–2008 in children and adults.^36^ The rates of methicillin‐resistant *S. aureus* (MRSA) abscesses, particularly in northern Australia, are increasing.^37^ The burden of abscesses disproportionately affects Aboriginal children. Skin abscesses are the most common reason for skin‐related hospitalisation in Aboriginal children in WA, accounting for almost half of all admissions to hospital for skin diseases, and the hospital length of stay is longer for Aboriginal children.^32^, ^33^

The prevalence of scabies documented in remote Aboriginal communities in the NT is high, ranging from 16.1% to 35%.^19^, ^21^, ^23^ There are less prevalence data available for other states: one study in the Pilbara documented a 5% prevalence rate,^22^ and another reported that 2% of all paediatric presentations to health clinics in the Western Desert region of WA between 2007 and 2012 were for scabies.^30^ This condition is a source of health inequity. In all children born in WA between 1996 and 2012, the highest disparity between Aboriginal and non‐Aboriginal hospital admission rates was in admissions for scabies among infants (incidence rate ratio, 417.0; 95% CI, 308.8–576.7).^32^

## Serious sequelae of untreated skin infections

The incidence of complications of skin infections has decreased for children from affluent populations but remains problematic in resource‐poor settings.^38^ These complications include invasive *S. aureus* and *S. pyogenes* infections and post‐infectious sequelae of *S. pyogenes*. Risk factors for the development of invasive GAS disease from a superficial infection include younger age, concurrent viral infection, household crowding, strain virulence, and impaired host immunity.^39^ While the key risk factor for acute rheumatic fever (ARF) and acute post‐streptococcal glomerulonephritis (APSGN) is untreated superficial GAS disease, it is not known why only a fraction of people infected with rheumatogenic GAS develop ARF.^40^

### Sepsis

The skin is an entry point for both bacteraemia and sepsis due to *S. pyogenes* and *S. aureus* infection.^39^ Incidence of *S. aureus* sepsis is tenfold higher for Aboriginal children compared with non‐Aboriginal children (46.6 *v* 4.4 per 100 000 children per year).^41^ Mortality for Aboriginal children admitted to the paediatric intensive care unit with *S. aureus* bacteraemia is threefold higher than for non‐Aboriginal children (47.6 *v* 15.9 per 100 000 children per year).^42^

### Skeletal infections

Osteomyelitis in children most commonly originates from haematogenous *S. aureus* and occasionally *S. pyogenes* seeding,^43^ and is often preceded by skin infection.^44^ The incidence of osteoarticular infection in children in northern Australia is among the highest in the world, with Aboriginal children (incidence, 90 per 100 000 per year) having rates tenfold higher than non‐Aboriginal children (incidence, 9 per 100 000 per year)^44^ of children living in high income countries, where the incidence is reported to be 3–13 per 100 000 per year.^43^

### Acute post‐streptococcal glomerulonephritis

Untreated GAS impetigo leads to APSGN after a latency period of 3–6 weeks.^45^ APSGN in Aboriginal Australian communities is linked to streptococcal impetigo, rather than pharyngitis, which is the source of most APSGN in temperate southern areas of Australia and most developed regions worldwide.^46^ In the NT outbreak of 2000, 87% and 40% of APSGN cases reported associated impetigo and scabies respectively, and *S. pyogenes* was isolated from 26% of cases, all of which were from skin sores.^47^ Although acute case fatality rates are low (< 2%),^48^ childhood APSGN increases later chronic kidney disease and dialysis risk in Aboriginal Australians.^49^

### Acute rheumatic fever and rheumatic heart disease

Symptoms and signs of ARF classically develop 2–3 weeks after the initial pharyngitis.^50^ The incidence of ARF in 5–14‐year‐olds in the NT is 150–380 per 100 000, and 2% of the Aboriginal population of the NT have rheumatic heart disease (RHD).^51^ However, some studies have reported that, in this setting, pharyngitis is rare, while impetigo is common, prompting the hypothesis that streptococcal skin infection may be implicated in ARF pathogenesis, either directly or via the pharynx.^52^, ^53^ In congruence with this hypothesis, molecular typing has many times reported a lack of differentiation in the Emm protein between throat and skin *S. pyogenes* strains.^54^ ARF occurs almost exclusively in Aboriginal people, with 94% of all 1776 cases between 2013 and 2017 being reported in Aboriginal Australians.^55^

## Treatment

Two Cochrane systematic reviews summarise the evidence for treatment of impetigo^56^ and scabies.^57^ However, they predominantly include studies from urban, outpatient, non‐epidemic settings. More recently, the evidence for the treatment of impetigo, scabies and fungal infections that relates to the heavy burden in Australian Aboriginal children in remote settings has been summarised and assessed using the GRADE approach^58^ (Box 4). Impetigo is effectively treated with topical antibiotics in non‐endemic settings. Either topical mupirocin or topical fusidic acid are recommended as first line therapy in guidelines^40^ and systematic reviews.^56^ However, in settings where the burden of impetigo is high, resistance may rapidly develop,^61^ and in these contexts, systemic antibiotics are recommended.^40^ For Australian Aboriginal children, the first line treatment for impetigo is oral trimethoprim–sulfamethoxazole for 3 days twice daily or 5 days once daily or an intramuscular injection of benzathine penicillin G.^40^ Alternatives to these regimens that have been shown to be effective in other endemic settings include oral amoxicillin or oral erythromycin.^62^ In scabies‐endemic settings, the treatment of comorbid scabies reduces the prevalence of impetigo.^63^ The number of sores is not used to stratify treatment.^10^

Box 4 Treatment advice and corresponding evidence strength rating as per the GRADE system*, 58 for skin and soft tissue infections in affected Australian Aboriginal children

**Table:** 

Condition	First line treatment	GRADE^58^	Second line treatment	GRADE^58^
Impetigo^59^	Peroral trimethoprim–sulfamethoxazole twice a day for 3 days or once a day for 5 days; or intramuscular benzathine penicillin G	1A	Peroral amoxicillin three times per day for 7 days; or peroral erythromycin four times per day for 7 days	2B
Scabies^59^	Topical permethrin on Day 1 and Day 8 for the index patient and household contacts	1A	Peroral ivermectin on Day 1 and Day 8 for the index patient who has failed treatment with topical permethrin within the preceding 4 weeks	1B
Cellulitis^60^	Intravenous β‐lactam for up to 2 days (for severe cellulitis only) followed by oral β‐lactam (eg, cephalexin four times per day for a total of 5 days)	1A	Peroral trimethoprim–sulfamethoxazole twice a day for 5 days; or peroral clindamycin three times per day for 5 days	1B
Abscesses^60^	Incision and drainage; followed by peroral clindamycin three times per day for 5 days; or peroral trimethoprim–sulfamethoxazole twice a day for 5 days	1A		

* **GRADE definitions:** 1A: Strong recommendation, applies to most patients without reservation. 1B: Strong recommendation, applies to most patients. 2B: Weak recommendation, alternative approaches are likely to be better for some patients under some circumstances.

First line scabies treatment in Australia is with topical permethrin.^12^, ^40^ Although effective, topical permethrin is oily and uncomfortable in hot humid climates^23^ and requires both a private space for application and functional health hardware to wash off. Hence, the public health approach to treat the index patient and all household contacts is not always adhered to. A study in remote Aboriginal communities in the NT documented treatment adherence rates of 80% (32/40) for the index case and 44% (193/440) for household contacts.^64^ Recent studies have confirmed the superiority of oral ivermectin for the treatment of scabies when used as mass drug administration for community‐wide control of scabies.^65^ For treatment of the individual and contacts, there is equal efficacy at 2 weeks for either topical permethrin or oral ivermectin.^66^ Ivermectin remains second line treatment in Australia and is only indicated if topical permethrin has been trialled and found to be ineffective within the past 4 weeks.^40^

There is a paucity of clinical trials on the treatment of cellulitis, with only 25 studies in 2488 adults — none of which reported on the same treatments, making comparisons difficult.^67^ Intravenous antibiotics may not be necessary.^67^ Severe cellulitis (rapidly spreading erythema, tenderness, lymphangitis, systemic symptoms) in children is treated with up to 2 days of intravenous antibiotics, usually a β‐lactam such as flucloxacillin, followed by up to 7 days of oral antibiotics.^68^ Cellulitis is usually caused by *S. pyogenes*, but confirming this microbiologically in a non‐purulent condition is challenging.^13^ In the MRSA era, a number of studies have compared oral antibiotic regimens in children and adults for the treatment of skin and soft tissue infections. Although rates of MRSA have increased from 7% to 24% of *S. aureus* isolates from remote community clinics in the NT between 1993 and 2012,^37^ clearance of *S. pyogenes* from impetigo lesions appears to be the key determinant of treatment success in this setting.^69^ While treatment with either oral clindamycin or trimethoprim–sulfamethoxazole is effective for cellulitis,^70^ MRSA‐active antibiotics may not be needed. When oral trimethoprim–sulfamethoxazole was added to cephalexin in comparison to cephalexin alone for treatment of uncomplicated cellulitis in two trials, there was no added benefit of the trimethoprim–sulfamethoxazole.^71^ β‐Lactams (eg, flucloxacillin or cephalexin) remain the treatment of choice for cellulitis, but trimethoprim–sulfamethoxazole and clindamycin are effective alternatives.^60^

An abscess is usually caused by *S. aureus*.^8^ Until recently, incision and drainage have been the treatment of choice for abscesses without the need for further antibiotics.^8^ Recent trials have confirmed the need for incision and drainage, with superiority demonstrated with the addition of either clindamycin or trimethoprim–sulfamethoxazole compared with placebo for up to 7 days after incision and drainage.^72^ For the treatment of abscesses in patients at increased risk of community‐acquired MRSA infection (including members of remote Aboriginal communities), incision and drainage followed by either trimethoprim–sulfamethoxazole or clindamycin for 5 days is recommended.^40^

## Challenges for the clinician

The normalisation of skin infections in high burden settings means that skin infections are often ignored, minimised or not treated.^31^ This increases the risk of post‐infectious complications, so it is necessary for all clinicians to be aware of the importance of skin infections. These infections are visible but often minimally symptomatic, and therefore, children and parents may not alert the clinician to this issue. A thorough skin examination looking for signs of skin infections is important in all Aboriginal children. When detected, treatment recommendations in the *National Healthy Skin Guideline* 10 or in the *Therapeutic Guidelines: Antibiotic* (www.tg.org.au) can be accessed for timely treatment guidance.

Prevention of early and complex skin infections is a high priority for families and health care providers. Impetigo declined considerably over the 2 years after the installation of a swimming pool in two remote WA communities,^22^ and although the observational nature of the data makes it difficult to attribute the effect entirely to the pools, a recent systematic review found that swimming pools are associated with a decrease in impetigo prevalence and severity.^73^ In urban settings, recurrent abscess requiring incision and drainage should prompt the clinician to consider the role of *S. aureus* decolonisation, a procedure that uses antiseptics and antibiotics to reduce the bioburden of skin pathogens and, in doing so, reduces the risk of recurrent skin infections.^8^ Attention to skin integrity, prevention of insect bites and regular handwashing^74^ will also help prevent skin infections.

Addressing the social determinants of health is ultimately needed for skin infections in Aboriginal children to reduce to parity with their non‐Aboriginal peers.^75^ Interventional research highlights that in high burden settings, individual treatment strategies without community‐level impacts are ineffective in reducing disease burden.^29^, ^63^ This is due in part to extensive community‐level transmission of impetigo, which sustains a high environmental load of microbial pathogens.^29^ The more immediate environment of the house also contributes to sustaining skin infection. Handwashing^74^ and scabies treatment with permethrin,^63^ both of which reduce impetigo prevalence, cannot be achieved without functioning health hardware, a long‐standing and widespread issue for communities where impetigo is common.^76^ Incorporation of high quality environmental health activities and partnerships with Aboriginal people, communities and service providers are needed to address this health disparity for Australian Aboriginal children. One example is the remote community swimming pool initiatives, which have improved skin health and general wellbeing in the Pilbara.^73^

## Conclusion

Impetigo, scabies, cellulitis and abscesses are skin infections that disproportionately affect Aboriginal children in Australia. More research is needed on the burden of disease in urban settings for Aboriginal children. In order to achieve healthy skin and healthy lives for all Australian children, randomised controlled interventional studies are required in order to eliminate the disparities in skin infection that contribute to morbidity and mortality.

## Competing interests

No relevant disclosures.

## Provenance

Commissioned; externally peer reviewed.
